# Limitations of effective medium models for tissue phantoms in the THz frequency range

**DOI:** 10.1038/s41598-024-70590-5

**Published:** 2024-10-03

**Authors:** Sonal Saxena, Ciaran Bench, Diksha Garg, Patric Boardman, Michal Mrnka, Harry Penketh, Nicholas Stone, Euan Hendry

**Affiliations:** Department of Physics and Astronomy, University of Exeter, Exeter, EX4 4QL UK

**Keywords:** Gelatine hydrogel, THz permittivity, Biological application of THz, Bound water, Raman spectra, Optics and photonics, Physics

## Abstract

The absorption of terahertz (THz) radiation by water molecules facilitates its application to several biomedical applications such as cancer detection. Therefore, it is critical for the THz technologies to be characterised with water content in a sample. In this paper, we analyse gelatine phantoms in the THz frequency range, with continuously varying hydration levels as they dry over time. Water molecules in close proximity to the protein molecule, termed ‘bound water’, feature properties different from the ‘free water’ molecules at larger distances. We find that a common model for predicting electromagnetic properties of phantoms and tissue samples, which assumes that only the free water varies with hydration while the bound water remains constant, does not agree well with measured results. To gain insight into this behaviour, we simultaneously measured the phantom in Raman spectroscopy, which shows a continuously varying concentration of bound water with hydration level. It follows from this investigation, that the permittivity contributions of neither the biomolecules nor water are expected to be linear with water density. This means that the often used, simple effective medium model will not be accurate for many biological tissues or phantoms.

## Introduction

The unique properties of terahertz (THz) radiation open up the potential for numerous biological applications. The non-ionizing and non-invasive nature of THz techniques renders them ideal for in-vivo biomedical studies^1,2^ like imaging of cancerous tissue^3^, examining tooth decay^4^, monitoring the healing of burns and wounds^5^, studying changes in corneal hydration^6^ and screening diabetic foot syndrome^7^. The emergence of several high-power THz sources^8^ and sensitive detectors^9^ in the recent years has enabled the realization of these concepts. The basis of all of these applications is the exhibition of high optical absorption by water molecules within the THz regime, resulting in limited penetration depths but high sensitivity to differences in water concentration^10^ enabling measurements of tissue pathology state.

Quantitative measurements of water concentration are needed to robustly inform clinical diagnoses, and consequently, the clinical adoption of THz sensing hinges on its ability to provide more than qualitative estimates of tissue hydration. To achieve this, a reliable model for the THz electromagnetic properties (here expressed as the permittivity) of tissues is required. Tissue “phantoms” can help here: materials designed to mimic the properties of human tissue. For THz frequencies, tissue phantoms are usually mixtures of proteins (such as gelatine) with water at densities comparable to biological tissues^11^. The dielectric properties of these phantoms are often described using the effective medium theory^12–14^, where the permittivity of individual components is used to calculate the composite value for the phantom. In a simplistic model, the phantom can be considered to be an effective medium composed of water molecules and long chain biomolecules like proteins and lipids. The large biomolecules do not have strong resonances in the THz frequency and are expected to feature a frequency-independent response, therefore, in the THz range the tissue response is expected to be dominated by water^13^. However, it is known that a significant fraction of the water molecules in such a mixture attach strongly to the protein molecules and form a shell-like structure around it. The motion of these water molecules, termed ‘bound water’, is constrained, and the permittivity component of these molecules is known to be smaller in the THz frequency range^15^. In contrast, the water molecules that are not bound to the protein biomolecules are expected to be closer in behaviour to the pure, liquid water and are termed as ‘free water’ - these are assumed to have permittivity values of liquid water in THz region^15,16^. The question is: how to combine the responses of protein, bound and free water components together in a suitable effective medium model?

In the most straightforward model commonly used in the literature to describe real tissues (for example in^12,13,16–18^), it is assumed that only the free-water component evaporates as the tissue sample dries. In this approach, referred to here as the constant background model, one measures the permittivity of a dried sample, and this measured value is presumed to account for the bound water and the large biomolecules, together describing the ‘biological background’ permittivity^13^. One then uses the biological background permittivity together with the permittivity of liquid water to describe the effective medium of the tissue/phantom, i.e. one assumes that the change in hydration of a tissue/phantom only corresponds to variation in free-water content while the bound-water content remains constant.

In this paper, we record the permittivity of a gelatine hydrogel with varying hydration levels and assess its suitability for use as a phantom for applications in the THz frequency range. The permittivity of gelatine phantom is measured periodically, as it dries, in a THz time-domain spectroscopy setup and is then compared with the model reported in the literature. The drying gelatine hydrogels are simultaneously studied using Raman spectroscopy to observe the bound-water content as hydration in the sample varies. We find that the constant background model does not agree well with measured results. From our Raman spectroscopy results, we observe a continuously varying concentration of bound water with hydration level, suggesting more complex models are necessary to fully account for this.

## Phantom preparation

Gelatine hydrogels are a good candidate for investigating the behaviour of tissue-mimicking samples in the THz frequency range. Gelatine is a long-chain molecule derived from collagen, which is the main structural protein present in various animal tissues including skin. Due to its biocompatibility, gelatine hydrogels have been researched and used in a wide variety of applications like drug delivery systems, wound dressing, and pharmaceutical capsules^19–21^.

Gelatine hydrogels have served as a model for biological tissues in a variety of spectroscopy and imaging studies, including in the THz frequency range. In previous studies^16,22^ gelatine phantoms with a range of water ratios were prepared and measured. For our measurements, we fabricate a hydrogel with high water content and record it drying over a period of time. We take the water concentration of the initial phantom to be 77% by weight of the mixture. This facilitates investigating the behaviour of sample over a wide range of hydration levels. The hydrogel samples are prepared using gelatine from porcine skin (48724-100G-F, Sigma-Aldrich). Water and gelatine are mixed in a beaker and heated at $60\,^\circ$C in a water bath. The mixture is then left to congeal overnight in a refrigerator in a covered mould to produce sub-millimetre thick phantoms.

## THz time-domain measurement

**Figure 1:**
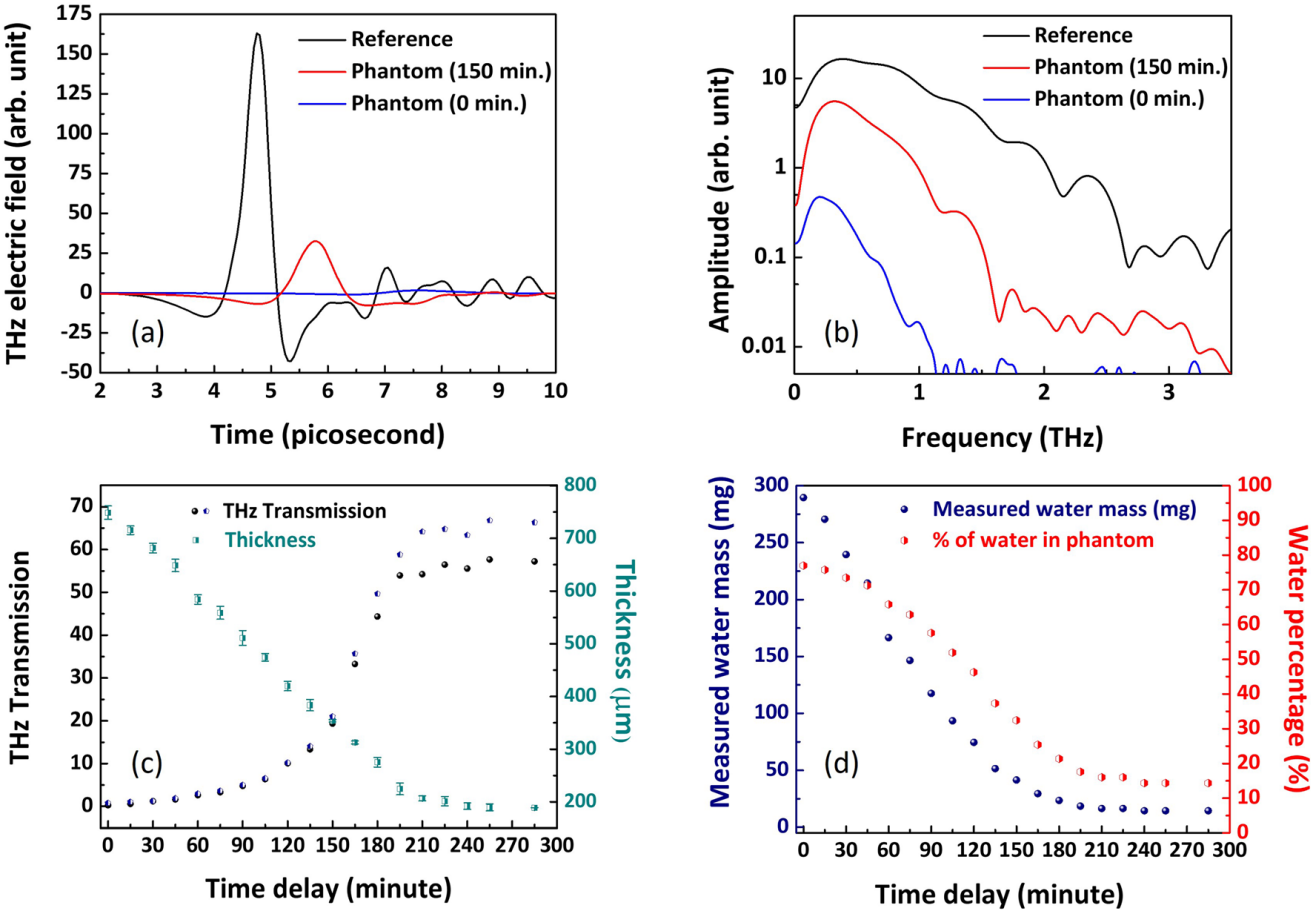
(**a**) THz time-domain signal and (**b**) spectrum for quartz slide as reference and phantom sample after ‘0’ and ‘150’ minutes of drying, (**c**) black graph is the ratio percentage of peak amplitudes of sample transmission signal with respect to the reference THz pulse and green graph shows the measured phantom thickness in microns. The blue plot shows the THz transmission coefficients for 600 GHz frequency. (**d**) Using the mass of phantom compared to mass of starting gelatine, water content is plotted as mass and percent by weight values for varying time delay.

The phantom samples are measured in THz transmission spectroscopy system. The THz spectrometer^23^ is driven by an ultrafast, Ti:sapphire laser emitting 100 fs duration pulses with 800 nm central wavelength at a repetition rate of 1050 Hz. The THz radiation in this setup is generated and detected using nonlinear effects of optical rectification^24^ and electrooptic sampling^25^ in ZnTe crystals. The gelatine phantom sample is mounted on a quartz slide and held vertically in the focal plane of the THz beam. The THz field that transmits through the drying phantom is recorded at regular intervals along with the reference time-domain signal with the quartz substrate only. The time-dependent electric field and spectrum transmitted through the reference and phantom sample drying for ‘0’ and ‘150’ minutes are shown in Fig. 1a and b respectively. The variation in transmission amplitude with time is plotted in Fig. 1c. The black graph is the ratio percentage of peak amplitudes of sample transmission signal with respect to the reference THz pulse and the blue plot shows the THz transmission coefficients for 600 GHz frequency.

**Figure 2:**
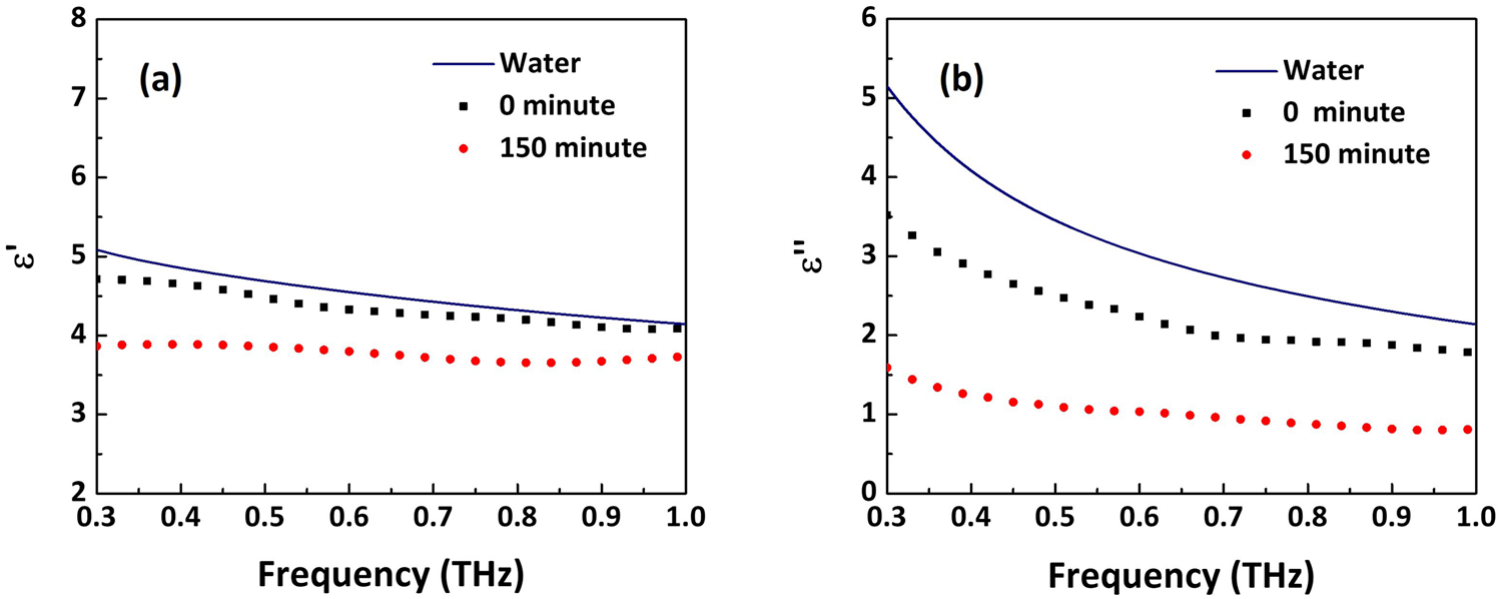
(**a**) Real and (**b**) imaginary part of the relative permittivity of the gelatine phantom at 0 and 150 minute time delay. The permittivity of pure water is plotted along with for comparison.

We also record both sample thickness and mass during the measurement run. Thickness is determined by a screw gauge with 0.001 mm resolution. For high water concentrations, the phantom sample is compressible and pressure from the micrometre measurement may lead to underestimation of the thickness^26^. The sample is therefore, also placed on a quartz slide of known thickness and imaged with high magnification, which allowed the thickness to be determined in a secondary, non contact method. The two thickness measurements with associated errors are combined to give the results shown in Fig. 1c. The mass of the phantom sample is also recorded with time using a weighing scale with 0.001 gm resolution and a chamber to protect from air draft. By recording difference in mass of the sample with time, and accounting for the gelatine mass used in sample preparation, the mass of water can be inferred for each time delay. This can then be converted to a percentage of total mass and is plotted in Fig. 1d.

From the THz time-domain signals and thickness measurements, complex relative permittivity, $(\varepsilon = \varepsilon ^{'} + i\varepsilon ^{''})$ of the phantom sample is calculated following ref.^27^. For individual frequencies, the difference in the experimentally observed transmission function and the calculated transmission derived from the thin-film transmission equation in ref.^27^ is minimized to obtain the relative permittivity of the sample. The real and imaginary parts of the calculated permittivity of the gelatine hydrogel after time delays of 0 and 150 minutes are plotted in Fig. 2a and b respectively. Note that we only plot the permittivity below 1 THz due to the low transmission through our phantoms for frequencies above this (see Fig. 1b). As observed in previous studies^22^, the imaginary part of permittivity shows stronger dependence on hydration level, as the difference in imaginary part of permittivities of the components (water and protein) is larger than the real part. The permittivity of pure water is shown in Fig. 2 as reference, plotted using Debye model values determined by Kindt and Schmuttenmaer^28^.

**Figure 3:**
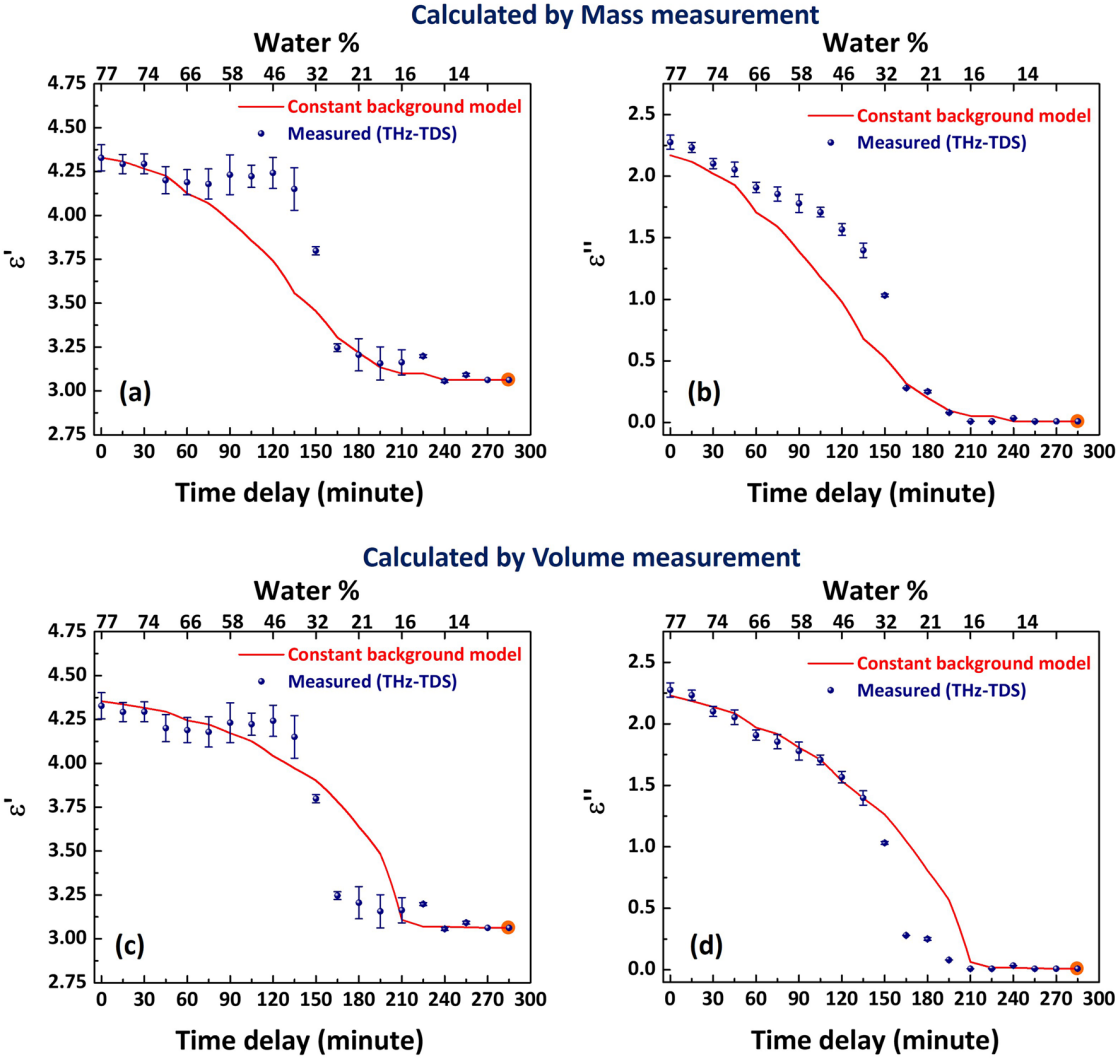
Real and imaginary parts of the relative permittivity measured for drying phantom at 0.6 THz are plotted as a function of time delay. Water percentage in the sample is shown on the top axes for reference. Corresponding permittivity values estimated from the mass measurement in the constant background model are shown in (**a**), (**b**), (**c**) and (**d**) show the comparison of measured and modelled permittivity values, when the fill fractions are calculated by variation in volume.

## Effective medium description

To describe the phantom sample as an effective medium using the constant background approach, contributions from two significant components, free water, and biological background^12^, are considered. The biological background is measured by fully dehydrating the material and measuring its dielectric properties. The dehydrated material is assumed to have all the free water evaporated and is left with bound water alone in addition to the large biomolecules. For all states of hydration, one can then in principle describe the effective permittivity if one simply knows the free-water content in the sample. To do this, one needs to know the fill fraction, i.e. the volume of a particular component divided by the total volume. A common approach to calculate this value is to use the measurement of water mass: by recording the additional water mass, and assuming the density of pure water, a volume can be associated with the free water in the sample^17,22^. Then, since the fill fraction of free water and biological background should sum up to unity, one can infer the fill fraction of both components.

To combine water and biological background components, we here use the Landau- Lifshitz-Looyenga (LLL) model as the shape of the solute is considered arbitrary^14,29^:

1 $$\begin{aligned} \root 3 \of {\varepsilon _{\textrm{eff}}} = f_{\textrm{free}} \root 3 \of {\varepsilon _{\textrm{free}}} + (1-f_{\textrm{free}}) \root 3 \of {\varepsilon _{\textrm{bb}}}. \end{aligned}$$

It has experimentally been shown that LLL is most suited for modelling biological tissue^30^. We plot the real $(\varepsilon ^{'})$ and imaginary $(\varepsilon ^{''})$ parts of the experimentally measured permittivity at an intermediate frequency in the THz transmitted spectrum at 600 GHz in Fig. 3. For implementing the effective medium model, the fill fraction and permittivity of the dehydrated sample are denoted as the biological background (‘bb’) - this is measured after 285 minutes of drying. As observed in Fig. 3, the permittivity of the phantom is no longer observed to change at this time. The effective permittivity is calculated by including the contribution of free water to the biological background for each time delay. The results for 600 GHz are plotted in Fig. 3 as ‘constant background model’. A comparison of the real (Fig. 3a) and imaginary (Fig. 3b) parts of permittivity calculated from the constant background model and measured experimentally is presented. Clearly, for the intermediate hydration levels expected for healthy and diseased real tissues^31^, such a simple effective medium model does not reproduce well the measured permittivity of our sample.

Since effective medium approximations intrinsically assume the density and permittivity of the components are independent parameters, it should not matter whether we calculate the volume fractions using mass or volume. In Fig. 3c and d, we plot permittivities calculated using volume fractions found using the measured sample thickness to determine the sample volume (note that we observe the variation in lateral dimensions is insignificant during the experiment). We see again that the model is not able to reproduce the measured values of permittivity for intermediate hydration levels. Moreover, the different behaviours of the model with component fill fractions calculated by mass and by volume point towards a deep problem in applying an effective medium: the density of the components appears to be changing with time. This contributes to the obvious difference in the measured permittivity values and the ones estimated by the constant background model. The difference is largest for 20 to 60% water, which is typical for many real tissues^31^. The conclusion of this work, therefore, is that this model does not adequately represent the true effective permittivity. The question is: why?

## Raman spectroscopy

**Figure 4:**
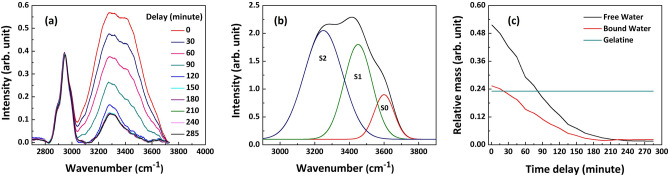
(**a**) High wavenumber region of the Raman spectrum measured for the drying gelatine phantom sample, (**b**) deconvolution of the Raman spectrum of pure water into three Gaussian components and (**c**) estimated relative mass of the bound water and free water along with gelatine.

We can gain some insight into the origin of the discrepancy in Fig. 3 using Raman spectroscopy. Raman measurements are taken using a homebuilt handheld surface probe, connected to a 785 nm laser^32^. The spectrum of drying gelatine phantom for varying time delays is shown in Fig. 4a. The Raman spectra shown have been background subtracted and normalised to the protein peak at 2943 cm^-1^. The water spectrum exhibits the hydrogen bonding action of OH vibrations, as Raman spectroscopy is sensitive to the molecular vibrations on the picosecond timescale^33^. It has been demonstrated in the literature that Raman spectroscopy can be applied for discriminating the different hydrogen bonds in water clusters^34^. Features of liquid water Raman spectra (calculated in Fig. 4b are composed of different hydrogen bonding arrangements. The simplest description of this multicomponent spectrum decomposes it into three Gaussian components (S2, S1 and S0) encompassing the most common bonding arrangements of water molecules^35^. As one progresses from the low wavenumber (S2) to the high wavenumber (S0) region the water molecules are less coordinated, forming fewer hydrogen bonds. The correlation of wavenumber and coordination can be clearly observed from the Raman spectra of ice^36^ and water vapour^37^. The spectrum shown in Fig. 4b, calculated using the peak parameters expected from liquid water using ref.^35^, is typical for that expected for ’free’ water. If water is ’bound’, it will typically be more coordinated (i.e. forming stronger, more permanent hydrogen bonds with the protein molecular structure^33^), and one would expect a greater oscillator strength in the S2 compared to S1 and S0 peaks. As can be seen in Fig. 4a, this is indeed what is observed as the sample is drying, with a gradual shift in relative oscillator strength to lower wavenumber.

Since the peak areas of S2, S1 and S0 represent the approximate number of water molecules in each of these coordinated states, we can use them to characterise the amount of ‘bound’ vs ‘free’ water in the sample at different stages of drying. For example, we see that even the phantom with the highest water density (labelled ’0 minutes’) has a greater proportion of S2 water compared to the liquid spectrum shown in Fig 4b. This tells us that even a water-rich phantom contains a sizeable fraction of ’bound’ water. As the sample is dried, the S2 peak becomes more dominant: as expected, the spectrum of a completely dehydrated sample after 195 min. is dominated by the S2 peak. However, we still have a tail of the Raman signal stretching to higher shifts, indicating that there is still some less coordinated, more ’free’ water left in the sample.

Using this approach, one can monitor the free and bound water contributions during drying. By comparing area ratio of the S2 peak to the total integrated spectral intensity found during peak fitting (using the peak parameters in ref.^35^) to that for liquid water, one can estimate the bound water fraction in the drying stages of our gelatine phantom (i.e. the peak ratios represented in Fig. 4b represent 100% free water, while a spectrum with oscillator strength only in the S2 state represents 100% bound water). Using the experimental weight measurements shown in Fig. 1d, estimated masses of bound and free water are calculated and presented in Fig. 4c. Note that, Raman intensity is a function of molecular density and Raman scattering cross-section, and we know that the Raman scattering cross-section of a molecule depends on its local field^38^ which is different for bound and free water. Since, here, we are directly using the intensity of deconvoluted peaks without considering the contribution of the Raman cross-section in estimating the volume fractions of bound and free water, this estimation method is at best approximate. Another factor to consider is the weak and broad NH peak at around 3300 cm^-1^ due to the gelatine molecule. As the phantom Raman spectra have been normalised for CH peak, the NH peak would result in a constant, small overestimation of the bound-water content. Nevertheless, very obvious and important deductions can still be drawn: it can be clearly seen from the results, that both the mass and the fill fraction of bound water change continuously as the phantom sample is drying. This observation suggests that the mixing of water and protein is much more complex than can be described by an effective medium model, which inherently assumes that the mixed material can be decomposed into the electromagnetic behaviours of the isolated components. The observation also agrees with previous results which show that the concentration of a gelatine molecule changes the surrounding environment of the water molecule, leading to the formation of differences in the water network with varying bond energy and strength^39^ as water concentration changes.

## Further discussion

The Raman results in the previous section clearly show that the mass of bound water and its fraction compared to the gelatine changes considerably during the drying process, i.e. the bound-water content does not remain constant with hydration in the hydrogel sample. With this observation, one cannot expect the background permittivity measured in a dehydrated sample (as done in refs.^12,13,16–18^) to be representative of the background permittivity of wet tissues/phantoms. One would need to take into account a different contribution for bound water permittivity, one that varies continuously with hydration level^39–41^. Moreover, with the mass of bound water continually changing during the drying process, one can expect complex molecular dynamics to change during drying.

**Figure 5:**
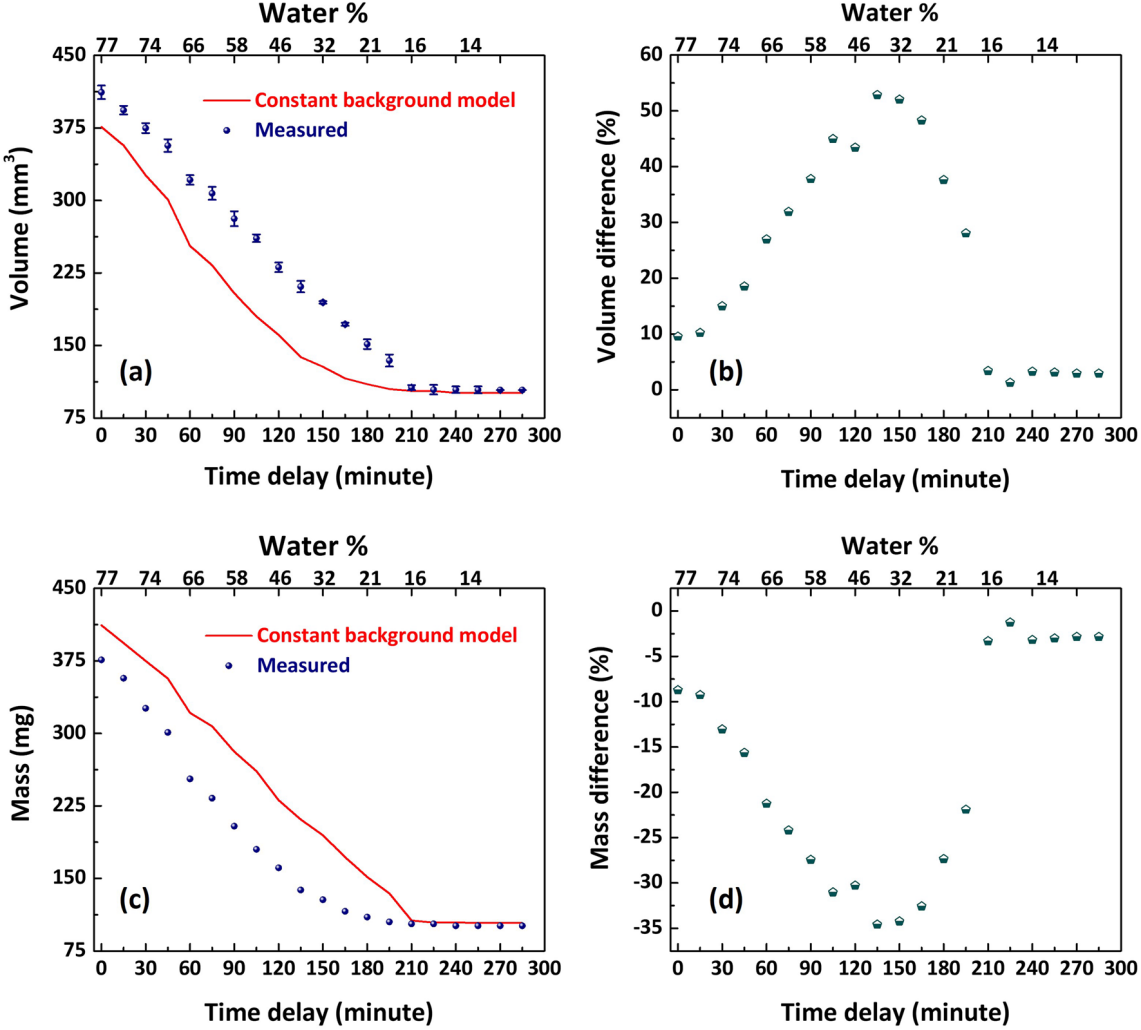
(**a**) Sample volume expected from free water mass variation in the constant background model is plotted along with the actual volume measured during the experiment (by measuring thickness and lateral dimensions). (**b**) The graph shows the volume percentage difference between the model and measurement. (**c**) The sample mass expected from the free water volume variation in the model is plotted along with the experimentally measured mass value. (**d**) The plot shows the percentage difference in mass between the model and the measurement.

We can gain some insight into this from the behaviour of the sample volume during the drying process. To interpret the difference in modelled and experimental results seen in Fig. 3a and b, we plot the behaviour of the sample volume measured with time delay in Fig. 5a as data points in blue colour. These values are proportional to the thickness measurement as we observe that variation in lateral dimensions is insignificant during the experiment. We can also estimate an expected volume assuming mixing of free water inherent for the effective medium model, using the measured mass of water and assuming the density of liquid water. This is plotted in Fig. 5a as the red graph for the model. A significantly larger measured volume than that estimated from the constant background model calculated using mass is evident. A maximum difference of > 50% for intermediate hydration values can observed from Fig. 5b, showing the percent difference in measured and modelled volume.

We can similarly compare our measured sample mass to that expected for an effective medium model calculated using measured sample volumes (i.e. the model results shown in Fig. 3c and d) again using the density of free water: this comparison is plotted in Fig. 5c. Again the difference between model and measurement is clear: the mass of the sample is significantly less than that required to describe the sample using a constant background model calculated using sample volume. The percent difference graph in Fig. 5d highlights the discordance in measurement and modelling.

Fig. 5a and c draw the same conclusion: the real density of the sample is considerably lower than expected for the simple mixing law which underlies the constant background model. This behaviour originates from the complex protein unfolding during hydration, which has been observed in hydrogel samples^41,42^. Similarly, the gelatine molecule swells in the presence of water, much more than expected from the addition of the water alone. From our data we see that this leads to the largest discrepancy for water concentrations in the range 20 to 60%.

It has been previously observed that effective medium approximations hold for mixtures of small molecules with water^43^. However, for larger molecules like proteins, the modelling is not that straightforward. The bound water and protein molecules undergo alterations with hydration level that are being studied^44^. Similar to the reports on protein hydration, a dramatic change in the permittivity is observed at around 150 minutes in Fig. 3. It has been postulated^45^ that such behaviour arises when the solute concentration increases to a value where the bound water hydration shells start overlapping. Such a sharp change in permittivity can also be explained on the basis of glass transition occurring in the gelatine hydrogel^42^. The swelling behavior in hydrogel is highly dependent on the degree of hydration. Dehydration leads to the transition from relaxed rubbery texture to unsolvated glassy consistency^39,42^.

## Conclusions

In this paper, we have shown that the direct application of a constant background effective medium model is not straightforward for gelatine phantoms. We identify two effects which are important. Firstly, using Raman measurements, we show explicitly that the bound water content in a sample is a function of hydration and not a constant. Secondly, this implies that the hydration behaviour of the molecules is complex, and we observe a significantly larger sample volume measured in experiment than is expected for a simple mixing law.

Such simple effective medium approximations are often used to determine water percentage from a measured permittivity in real tissues^13,17^. In such cases, the error in the extracted water percentage can be expected to be large, even up to a factor of two for the most divergent predictions around  50% water. We have identified two factors that obstruct such quantitative analysis of water content in a sample. Firstly, the hydration-dependent density of bound water will lead to a water contribution to the overall permittivity that is hydration dependent. Secondly, due to this, the density of the hydrated protein can vary significantly with hydration level^42^. It follows from these effects that the permittivity contributions of neither background nor water are expected to be linear with water density. This means a constant biological background permittivity model will not be accurate for many phantoms or indeed biological tissues, and water densities extracted using such models can be expected to have large systematic errors associated. Moreover, we argue that effective medium models in general are of limited use to many biological systems: at their heart lies a hypothesis that the total permittivity of the system can be described as a linear sum of components. When the components themselves interact, with changes to one permittivity component affecting the permittivity of the other in a way that they are not independent parameters, such a description loses meaning.

## Data Availability

The datasets analysed during the current study are available from the corresponding author upon reasonable request.
